# Mediterranean Lavenders from Section *Stoechas*: An Undervalued Source of Secondary Metabolites with Pharmacological Potential

**DOI:** 10.3390/metabo13030337

**Published:** 2023-02-24

**Authors:** Joana Domingues, Fernanda Delgado, José Carlos Gonçalves, Mónica Zuzarte, Ana Paula Duarte

**Affiliations:** 1 Plant Biotechnology Centre of Beira Interior (CBPBI), 6001-909 Castelo Branco, Portugal; 2 Health Sciences Research Centre (CICS), University of Beira Interior, 6200-506 Covilhã, Portugal; 3 Polytechnic Institute of Castelo Branco-School of Agriculture (IPCB-ESA), 6001-909 Castelo Branco, Portugal; 4 Research Centre for Natural Resources, Environment and Society, Polytechnic Institute of Castelo Branco (CERNAS-IPCB), 6001-909 Castelo Branco, Portugal; 5 Coimbra Institute for Clinical and Biomedical Research (iCBR), Faculty of Medicine, University of Coimbra, 3000-548 Coimbra, Portugal; 6 Center for Innovative Biomedicine and Biotechnology (CIBB), University of Coimbra, 3000-548 Coimbra, Portugal; 7 Clinical Academic Centre of Coimbra (CACC), 3004-531 Coimbra, Portugal; 8 Faculty of Health Sciences, University of Beira Interior, 6200-506 Covilhã, Portugal

**Keywords:** *Lavandula pedunculata*, *Lavandula stoechas* subsp. *luisieri*, *Lavandula stoechas* subsp. *stoechas*, *Lavandula viridis*, chemical profile, biological activities

## Abstract

Globally, climate change and wildfires are disrupting natural ecosystems, thus setting several endemic species at risk. The genus *Lavandula* is widely present in the Mediterranean region and its species, namely, those included in the section *Stoechas,* are valuable resources of active compounds with several biological assets. Since ancient times lavenders have been used in traditional medicine and for domestic purposes. These species are melliferous, decorative, and essential oil-producing plants with a high economic interest in the pharmaceutical, flavor, fragrance, and food industries. The essential oils of *Lavandula* section *Stoechas* are characterized by high amounts of 1,8-cineole, camphor, fenchone, and specifically for *L. stoechas* subsp. *luisieri* one of the major compounds is *trans*-α-necrodyl acetate. On the other hand, the diversity of non-volatile components like phenolic compounds, such as phenolic acids and flavonoids, make these species an important source of phytochemicals with pharmacological interest. Rosmarinic, caffeic, and salvianolic B acids are the major phenolic acids, and luteolin and eriodictyol-*O*-glucuronide are the main reported flavonoids. However, the concentration of these secondary metabolites is strongly affected by the plant’s phenological phase and varies in *Lavandula* sp. from different areas of origin. Indeed, lavender extracts have shown promising antioxidant, antimicrobial, anti-inflammatory, and anticancer properties as well as several other beneficial actions with potential for commercial applications. Despite several studies on the bioactive potential of lavenders from the section *Stoechas*, a systematized and updated review of their chemical profile is lacking. Therefore, we carried out the present review that gathers relevant information on the different types of secondary metabolites found in these species as well as their bioactive potential.

## 1. Introduction

**Figure 1:**
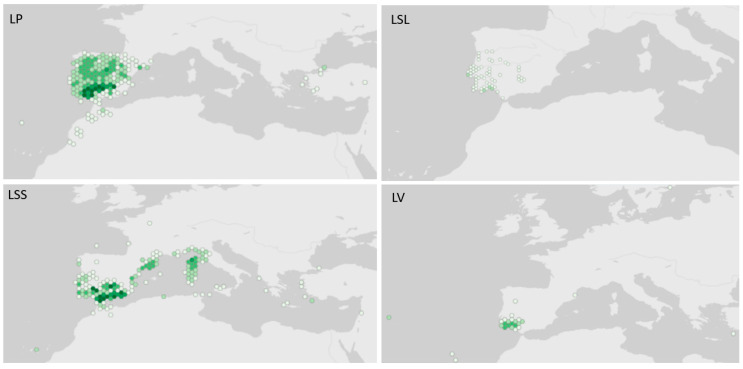
Native distribution of *Lavandula* species from the *Stoechas* section, using georeferenced data from Global Biodiversity Information Facility (in GBIF Secretariat, 2023). GBIF Backbone Taxonomy. LP: *L. pedunculata* (Mill.) Cav., available online: https://www.gbif.org/species/7307760; LSL: L. *stoechas* subsp. *luisieri* (Rozeira) Rozeira, available online: https://www.gbif.org/species/7660687; LSS: *L. stoechas* subsp. *stoechas*, available online: https://www.gbif.org/species/7307711; LV: *L. viridis* L’Hér., available online: https://www.gbif.org/species/3890777; accessed via GBIF.org on 13 January 2023, 17:08 PM. Inset presents the geographic context of *Lavandula* species from the *Stoechas* section distribution area. Dark green means higher occurrence.

The Mediterranean region is described as a biodiversity hotspot due to its favorable geographical location that enables the growth of several unique plant species, including endemisms. However, it is also considered one of the regions with habitat loss, largely due to the climate changes and the high risk of fires associated with this region. The genus *Lavandula* belongs to the Lamiaceae family and includes around 39 species distributed from the Mediterranean region to India, and North Atlantic Islands (Figure 1) [1]. This genus includes wild taxa, hybrids, and several cultivars and field varieties. *Lavandula* species are associated with the syntax of Cisto-Lavanduletae, which includes thermo- to supra-Mediterranean dry, semi-arid sub-humid secondary scrub communities producing aromatic and medicinal compounds [2]. The Lamiaceae family has a cosmopolitan distribution, and these species can grow in very dry and hot climates, contributing to the adaptation of plants to extreme conditions of water stress and high temperatures, which appears to increase the production of secondary metabolites [3].

*Lavandula,* commonly known as lavender, is an “ancient world” genus; although, more recently, some species have been widely cultivated and are highly appreciated in perfumery and cosmetic products. Based on the most recent phylogenetic study carried out on the *Lavandula* genus, Moja et al. distinguished six sections, namely, *Lavandula*, *Dentata*, *Stoechas*, *Pterostoechas*, *Chaetostachys*, and *Subnudae* [4]. In the present review, we focus on the *Stoechas* section, which includes three species, *Lavandula pedunculata* (Mill.) Cav., *Lavandula stoechas* L. subsp. *luisieri* (Rozeira) Rozeira, *Lavandula stoechas* L. subsp. *stoechas*, and *Lavandula viridis* L’Hér., all endemic to the Mediterranean region. In most studies, the authors do not mention with which subspecies they work. For example, in *L. pedunculata*, only four studies referred to the subspecies *sampaiana* (Chaytor) Franco [5,6,7,8]. Further, regarding *L. stoechas* subsp. *stoechas,* some authors only refer to *L. stoechas* L. These species grow in the semi-arid and rather inhospitable soils in this region, which reveals their strong resistance and suitability to cultivation with inexpensive maintenance of the soil. Only three *Lavandula* species are cultivated for the commercial production of their essential oils, namely, *Lavandula angustifolia* Mill. (fine lavender or English lavender), *Lavandula* x *intermedia* Emeric (lavandin), and *Lavandula latifolia* Medicus (spike lavender) [9]. According to several authors, in terms of morphology, the section *Stoechas* is distinct from the others due to its large and colored apical bracts located at the apex of the flower spikes [1,10]. However, several taxonomic modifications have been suggested over the last few years, mainly due to the high morphological variability and hybridization ability among these species. Therefore, for an accurate description and classification, it is important to consider the occurrence of polymorphisms and chemotypes [11]. A chemotype is a chemically distinct entity in a plant with differences in the composition of the secondary metabolites. Minor genetic and epigenetic changes with little or no effect on morphology or anatomy may produce large changes in the chemical profile [12]. Regarding wild taxa classification and identification, uncertainties, and doubts are very frequent due to hybrid forms or polymorphisms. Indeed, the taxonomical classification of lavender species is quite controversial among the scientific community, and several taxonomic changes have been advised over the years. The high polymorphism among these species influences the variability of their chemical components, which also depends on several abiotic factors including geographical and climatic conditions [13]. Importantly, the presence of some chemical compounds can be a chemotaxonomic distinctive feature and, therefore, be used to distinguish species [14,15]. Aprotosoaie et al. demonstrated in their chemical review of essential oils of the *Lavandula* genus that only five species, two of them being hybrids, have ISO standards, or a standard chemical profile for the essential oils included in the European Pharmacopoeia [16]. Therefore, knowledge of the chemical profile of each species can be decisive for their correct identification, valorization, regulamentation, and industrial exploitation.

Species from the section *Stoechas* are aromatic perennial shrubs that have been reported since ancient times due to their medicinal, fragrance, and culinary uses. These species are rich in valuable essential oils and other secondary metabolites, including phenolic compounds, with several beneficial properties. Indeed, ethnobotanical studies have shown the popular uses of some of these species for the treatment of digestion, headaches, heartburn, blood circulation, as a sedative, antidermatitis, nasal decongestive bronchitis, and asthma [17,18]. In recent years, many of these traditional uses have been validated in scientific-based studies, and several volatile and non-volatile compounds have been identified as promising lead compounds. For example, antibacterial, antifungal, anti-inflammatory, analgesic, carminative, antidepressant, and sedative properties have been pointed out, thus highlighting the beneficial potential of these species [19,20,21,22,23]. Moreover, in several countries, for example, Portugal, the number of distilleries has increased mainly due to the knowledge and interest of consumers in these natural products and their exceptional properties for human health. Currently, many companies collect the raw material in the wild, but some of them cultivate these species and, gradually, the uncultivated fields come to life with new aromatic crops. Plant harvest in the wild should be avoided as it can lead to biodiversity loss. Furthermore, the destruction of the species’ habitats also occurs as a result of fire disasters and due to climate changes, which can lead to the fragmentation of plant populations, thus decreasing their size and number and, consequently, resulting in genetic erosion. Valuing these plant species can contribute to the revitalization of poor soils and damaged areas by wildfires that have devastated the Mediterranean region in the last few years. Therefore, it is important to properly identify and valorize these ancient and endemic species through an accurate chemical characterization and biopotential validation.

## 2. Methodology

The present literature review on *Lavandula* section *Stoechas* covers several topics including secondary metabolites found in essential oils and other extracts and their biological activities. This review was performed from several scientific databases, such as Google Scholar, Science Direct, Springer Link, Wiley Online Library, PubMed, and ResearchGate, and combined the following keywords: “*Lavandula* essential oil”, “*Lavandula* extracts”, “*Lavandula* chemical composition”, and “*Lavandula* biological activity”. The literature review on the chemical characterization of essential oils or other extracts was compiled for each species, as follows: *L. pedunculata* (9 articles), *L. stoechas* (30 articles), *L. stoechas* subsp. *luisieri* (16 articles), and *L. viridis* (6 articles). Regarding pharmacological and other biological activities, generally, the same article has more than one biological activity reported, and a few of them reported more than one species. From 163 consulted references, 95% correspond to scientific articles, 3% refer to books, and 2% are books chapter, all published between 2002 and 2021.

## 3. Secondary Metabolites from *Lavandula* Section *Stoechas*

Secondary metabolites can be obtained from fresh or dried plant parts and at different phenological stages. To preserve the chemical composition of the extracts and their metabolites, appropriate extraction methods should be considered. Currently, the extraction method recommended by the European Pharmacopoeia to obtain essential oils is through distillation, which can be divided into several types such as water or hydrodistillation (HD), steam distillation (SD), or by the expression for citrus fruits [24]. The distillation method is the only recognized reference process to obtain essential oils (excluding those from citrus fruits) and presents several advantages such as low cost, reduced need for labor, and a greater number of plants that can be processed at the same time. Nevertheless, other non-volatile extracts can be obtained with different types of solvents including water, methanol, ethanol, or non-polar solvents, thus enabling the extraction of non-volatile compounds such as phenolic acids, flavonoids, triterpenes, and coumarins [8,25,26]. However, these methods have some disadvantages, such as the formation of thermally degraded by-products, the presence of solvent residues, and lower yields [27]. More recently, supercritical fluid extraction (SFE) has been reported as the most natural extraction procedure to obtain more pure/specific compounds. The product obtained by supercritical extraction is nominated as a volatile oil to distinguish it from the essential oil produced by HD or SD. SFE can be considered as an alternative process compared to traditional methods, due to the lower operating temperatures and absence of solvent residue in the product. Nevertheless, this technique has high equipment costs and requires operating expertise (fluid, pressure, temperature, extraction time, and plant material/solvent ratio conditions). Moreover, the presence of waxes in the volatile oil has been reported as a disadvantage [28,29].

Regarding the section *Stoechas*, extracts from the aerial parts of *L. stoechas* subsp. *luisieri* were obtained with an average yield of 1.4% and 0.4%, for SFE (CO_2_) and HD extraction, respectively [25]. The SFE was also performed in *L. viridis* with values of 3.4% and 0.5% for SFE (CO_2_) and HD, respectively [30]. In both reports, although the extraction yield was higher with the SFE, it seems that the diversity of some chemical compounds was lost, when compared to the HD method, thus highlighting the relevance of the approved extraction method for the quality of the extracts. Next, the main type of compounds found in species from the section *Stoechas* (essential oils and non-volatile compounds) is presented with a brief introduction regarding chemical composition and main compounds, relevant features, biosynthesis, and bioactive properties.

### 3.1. Essential Oils

Terpenes or terpenoids are the most important constituents of essential oils, with more than 40,000 individual compounds already identified in the Plant Kingdom. These secondary metabolites play an important role in the interactions of plants with the environment and ecosystem, namely, in plant defense and insect attraction for pollination. For example, some compounds are directly involved in pollinator attraction (linalool, linalyl acetate), act as mediators in plant defense against stress (camphor and 1,8-cineole), or as insect repellents (necrodol) [13].

The biosynthesis of terpene compounds starts with two interconvertible molecules with five-carbons units: isopentenyl diphosphate (IPP) and its allylic isomer dimethylallyl diphosphate (DMAPP). These precursors are synthesized from different pathways: the mevalonic acid (MVA) and the methylerythritol phosphate (MEP) pathways [31]. Monoterpenes are mainly produced in the MEP pathway, whereas sesquiterpenes have an origin in the MVA pathway [32]. Beyond being compartmentally separated (MVA in the cytosol, peroxisomes, and endoplasmic reticulum; and MEP in plastids), these biosynthetic pathways are interconnected by a metabolic “cross-talk” [33]. Most common terpenes, called regular terpenes, are produced by the sequential head-to-tail addition of DMAPP to IPP, while the less common irregular terpenes are produced by the non-head-to-tail joining of the two building units or by rearranging a regular structure [34].

In *Lavandula* species of the section *Stoechas*, the terpenoids, mainly mono- and sesquiterpenes are the most studied due to their abundance in essential oils. The oxygenated derivatives of terpenoids are also abundant, namely, several irregular monoterpenoids. Beyond their relevance in plant–environment, plant–plant, or plant–insect interactions, these compounds also present interesting fragrance and flavor features. The medicinal properties reported for *Lavandula* species are mainly due to their essential oils, which also contribute to the aroma of these species. Lavender essential oil is a colorless to pale yellow liquid with a characteristic sweet, fresh, floral-herbaceous with a slight balsamic-woody undertone odor [1]. In perfumery and cosmetic industries, the most appreciated lavender oils are those with a high content of linalool and its esters, and low content in camphor, whereas the essential oils rich in camphor, are mostly appreciated in aromatherapy and phytotherapy due to their therapeutical properties [35]. The specific aroma in addition to diverse biological properties has considerably improved the industrial value of some of these species in several areas such as medicine, cosmetics, perfumery, and also as food preservatives [36]. The essential oils are produced and stored in specialized secretory structures distributed along different parts of the plant and the secretory stage depends on the phenological stage of the plant [37]. Lavender essential oils are produced and accumulated in glandular (capitate and peltate) and non-glandular trichomes, similar to what has been described for *Rosmarinus* [3]. All types of glandular trichomes are involved in essential oil production, except capitate type I trichomes. Stellate non-glandular trichomes and glandular trichomes of different types: peltate, capitate type I, and capitate type II have been reported in *L. pedunculata*, *L. stoechas* subsp. *luisieri*, and *L. viridis*. Furthermore, bifurcated trichomes were found in *L. pedunculata* and *L. viridis* [38,39].

The essential oils of section *Stoechas* are generally characterized by the presence of monoterpenes hydrocarbons, oxygenated monoterpenes, and sesquiterpenes. In some cases, non-terpenic compounds also occur, namely, phenylpropanoids. The first ones are light and very volatile molecules also called “top notes” by the perfumery industry, while the sesquiterpenes are less volatile, showing a high potential for stereochemical diversity and also responsible for stronger odors [27]. The quality and chemical composition of essential oils depend on various factors, such as genotype, botanical organ, phenological stage of the plant, environmental conditions, harvest time, and also extraction methods used [38,40,41]. Even in the same species, essential oils usually have a variable chemical composition due to intrinsic (seasonal, ontogenetic, and genetic) and extrinsic (geographical, ecological, and environmental) factors [16,41].

Studies on the composition of *Lavandula* essential oils from the section *Stoechas* reported so far are summarized in Table 1. The values presented vary from the lowest to the highest concentration found in the literature. For all species, most of the compounds found in their essential oils are oxygenated monoterpenes (33–87% of the total identified compounds), followed by oxygenated sesquiterpenes (0.3–20%), and by monoterpene hydrocarbons (0.1–19%). The absence or low amount of some compounds or even the significant presence of distinct compounds in plants from the same species may allow the identification of distinct chemotypes. Overall, several studies on *L. pedunculata* from the Mediterranean region reported camphor, fenchone, and 1,8-cineole, as the major compounds of its essential oil [5,38,39,42,43,44]. Studies from the Morocco region reported a chemical profile with camphor (46–53%) as a major compound, followed by fenchone (1.3–13%), α-pinene (2–11%), camphene (5.7–6.1%), and 1,8-cineole (0–6.5%) [43,44]. In some cases, the concentrations of these major compounds varied, allowing the identification of chemotypes. Indeed, Zuzarte et al. analyzed the chemical profile of *L. pedunculata* essential oils from the North and Center of Portugal and pointed out some differences in the chemical polymorphism with plants from the North being rich in 1,8-cineole/camphor (24%/32%) and plants from Central Portugal presenting high amounts of fenchone (49%) [39].

Concerning *L. stoechas* essential oils, some studies reported the chemical composition of plants from Tunisia, Turkey, Iran, Morocco, Algeria, Greece, and Spain [46,47,48,49,50,51,52,53,54,55,56,57,58,59,60,61]. The most abundant and common chemical compounds reported were camphor (72%), fenchone (68%), 1,8-cineole (61%), α-pinene (23%), β-pinene (14%), α-terpineol (13%), and camphene (11%). Nevertheless, these studies failed to identify the subspecies considered. Carrasco et al. studied the chemical profile of the *L. stoechas* essential oils from plants growing in the southeast of Spain, from the supra-Mediterranean and upper meso-Mediterranean bioclimatic zones. The authors revealed differences between essential oils concerning the presence and number of minor compounds, such as linalool, bornyl acetate, thymol, myrtenyl acetate, and viridiflorol [55]. In this sense, different chemical profiles have also been reported in locations outside the Iberian Peninsula and some essential oils showed an uncommon chemical profile. For example, the essential oil of Sardinian plants showed significantly unusual concentrations of bornyl acetate (6.2%), thymol (3.1%), and guaiol (1.7%) [62]. Karabagias et al. also reported an uncommon chemical profile of *L. stoechas* essential oil from Greece whose main compounds were thujone (32%) and myrtenyl acetate (8%) [60]. From the African region, linalyl acetate (64%) and linalool (20%) were the major compounds found in Tunisian plants, and from Moroccan plants, an unusual profile with high concentrations of 10s,11s-himachala-3(12),4-diene (24%), cubenol (16%) and methyl eugenol (6%) was reported in *L. stoechas* essential oil [52,86].

As previously mentioned, *L. stoechas* subsp. *stoechas* and *L. stoechas* subsp. *luisieri* are two subspecies of *L. stoechas*, with very similar morphological features; however, they are very distinctive in what concerns the chemical composition of their essential oils. Although the authors of the above-referred studies did not specify on which subspecies they were working, by observing the chemical profile reported in their studies, it seems that the characterization refers to *L. stoechas* subsp. *stoechas* essential oil, due to the presence of characteristic major compounds. Indeed, *L. stoechas* subsp. *stoechas* essential oil has been characterized by high concentrations of fenchone (40–71%), camphor (12–47%), and 1,8-cineole (15–18%) [77,78,79]. Nevertheless, Gören et al. reported a distinct chemical profile for Turkish *L. stoechas* subsp. *stoechas* with pulegone (40%), menthol (18%), and menthone (13%) being identified as major compounds, and common compounds such as fenchone, camphor, and 1,8-cineole not being detected [80].

According to Upson and Andrews, *L. stoechas* subsp. *luisieri* or *L. luisieri* (denomination considered by some taxonomical classifications) is only found in the Iberian Peninsula, namely, in the southwest of Spain and Portugal [1]. Most of the studies on *L. stoechas* subsp. *luisieri* refer to plants collected in Portugal, contrasting with a few studies from Spanish plants. Besides the very small morphological differences between subsp. *stoechas* and subsp. *luisieri*, the latter showed a very peculiar and distinctive chemical profile compared to other species of the section *Stoechas* and to plant species in general. Interestingly, in this species, irregular monoterpenoids with cyclopentenic structures, namely, necrodane derivatives, are present in their essential oils. García-Vallejo found these compounds for the first time in the essential oil of *L. stoechas* subsp. *luisieri*, with α-necrodol and α-necrodyl acetate reported as the main compounds [87]. These kinds of compounds were discovered by Eisner and Meinwald in the defensive secretion of *Necrodes surinamensis* [88] and, recently, they were found exclusively in *L. stoechas* subsp. *luisieri* and *Evolvulus alsinoides* L. essential oils [89]. The necrodane monoterpenoids can be considered a chemotaxonomic marker of this subspecies [14,36]. As far as we know, the biosynthesis of these irregular monoterpenoids is still unclear. Due to their very unusual structure, some synthetic routes have been suggested for *trans*-necrodol and its isomers. Figadère et al. suggested that the necrodol skeleton could be generated from geranyl diphosphate from the typical head-to-tail 1′-4-linkage of DMAPP and IPP via the isocamphane skeleton [90]. Vacas et al. confirmed the presence of necrodane compounds in the sex pheromones of *Delottococcus aberiae* (mealybug), which are mainly composed of irregular terpenoids that show non-head-to-tail 1′-2 linkages between the isoprene units. The authors suggested an alternative biosynthetic pathway to the necrodol skeleton via a lavandulyl cation [91]. Concerning the main compounds of *L. stoechas* subsp. *luisieri* essential oil from the Iberian Peninsula, camphor (1.1–74.4%), *trans*-α-necrodyl acetate (1.8–48.2%), 2,3,4,4-tetramethyl-5-methylcyclopenten-2-enone (2–38%), fenchone (0.1–22%), and 1,8-cineole (1.3–21%) have been reported as the major constituents [5,14,19,20,25,36,63,70,71,72,73,74,75,76]. In most studies of plants from Portugal, the main chemical compounds present in the essential oil of *L. stoechas* subsp. *luisieri* are *trans*-α-necrodyl acetate followed by 1,8-cineole, fenchone, and camphor [5,20,25,36,72,73,74,75,76]. In Table 1, a high camphor concentration (74%) is shown; however, this value was only found in plants from Spain, in which the essential oil revealed some differences in comparison to plants from Portugal, where the main compound was *trans*-α-necrodyl acetate [25,36,72,74,75,76]. Although most studies have pointed out the presence of irregular monoterpenoids in this species, namely, necrodane derivates, chemical variability seems to be very common among populations. Zuzarte et al. found considerable differences in the chemical composition of essential oils between plants in central and southern Portugal. In the first region, the essential oil was characterized by the presence of *trans*-α-necrodyl acetate (17%), followed by *trans*-α-necrodol (7%) and 1,8-cineole (6%), while the main compounds of the essential oils from southern plants were 1,8-cineole (34%) and fenchone (18%), and the concentration of necrodane compounds was very low (around 3% of *trans*-α-necrodyl acetate) [36]. Domingues et al. reported a high content of *trans*-α-necrodyl acetate (27%), *trans*-α-necrodol (13%), lavandulyl acetate (7%), and linalool (6%) in the flowering phenological stage, while the essential oil of the dormancy phase had a lower necrodane content; however, 5-methylene-2,3,4,4-tetramethylcyclopenten-2-enone (11%), fenchone (6%), 1,8-cineole (5%), and camphor (3%) concentrations were higher than those observed in the essential oil obtained from the flowering stage [19]. Regarding the reported studies, differences in the concentration of necrodane compounds are frequent, and it has been reported that essential oils with higher amounts of these compounds have more potent biological activities [25,36,72].

Due to the morphological variability and hybridization capacity of *L. stoechas* in Portugal, the taxonomy of the genus *Lavandula* has undergone several taxonomic changes. Specifically for *L. stoechas* subspecies, the variability found between the concentrations of the main components suggests the presence of several chemotypes [11]. An interesting approach was reported by Guitton et al., who studied qualitative and quantitative changes in terpenes at different maturing stages of lavender flowers (unopened flowers, opened flowers, and faded flowers). The opened flowers presented linalyl acetate and some sesquiterpenes, as the main compounds, which suggests that they can act as attractive molecules for pollinating insects. While the unopened and faded flowers revealed 1,8-cineole, ocimene, limonene, linalool, and terpinen-4-ol as the main compounds, which indicates a repellent action to protect immature flowers and seeds from harmful insects. Thus, differences found in the chemical composition between different species refer essentially to genetic and evolutionary factors [92].

**Figure 2:**
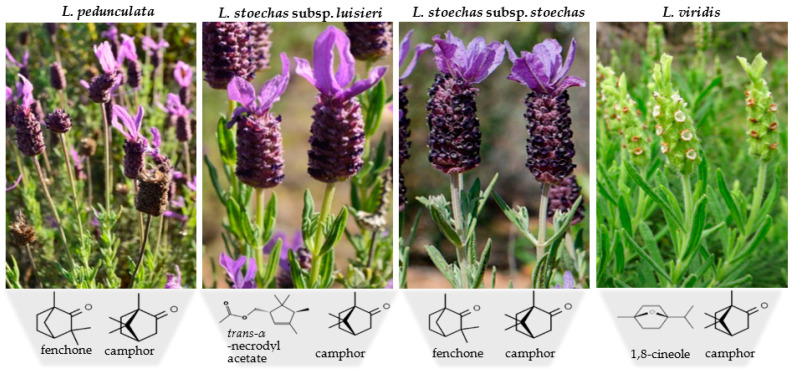
Morphological traits of *Lavandula* section *Stoechas* species and its main compounds. *L. stoechas* subsp. *stoechas* and *L. viridis* photographs were downloaded from Flora de Portugal Interactiva. (2014). Sociedade Portuguesa de Botânica (https://www.flora-on.pt, accessed on 25 October 2021). The chemical formulas were obtained in Chemical Structure Search–ChemSpider (https://www.chemspider.com/StructureSearch.aspx, accessed on 25 October 2021).

Regarding *L. viridis* essential oil from the south of Portugal, similar chemical profiles have been reported in different studies, suggesting a high homogeneity of this volatile extract, with the main compounds being 1,8-cineole (21–74%) and camphor (3–32%), followed by α-pinene (0.3–9%) and camphene (0.1–8%) [30,84]. In contrast to the other species, the absence of fenchone and lavandulyl acetate was verified in all reports on the essential oil of this species, as well as the low presence of sesquiterpenic compounds. Figure 2 shows the species included in section *Stoechas* and their respective main compounds.

As a unifying feature, all species from the section *Stoechas* present in their essential oil composition considerable concentrations of camphor and 1,8-cineole. Camphor is a monoterpenoid ketone with two enantiomeric forms: (1S)-(-)- and (1R)-(+)-camphor. Both forms have a similar camphoraceous fragrance; however, the stereochemistry effects on the biological properties are still unknown. In plants, camphor is produced by the cyclization of geranyl diphosphate by the enzyme (+)-bornyl diphosphate synthase generating (+)-bornyl diphosphate. The hydrolysis of (+)-bornyl diphosphate then originates (+)-borneol by the action of bornyl-diphosphate diphosphatase and the (+)-borneol dehydrogenase oxidates (+)-borneol to (+)-camphor [93]. Camphor can also be synthesized from α-pinene obtained by the distillation of turpentine oil (the resinous exudate obtained from coniferous trees) mainly from *Cinnamomum camphora*. Several pharmacological properties are reported for camphor, namely, antimicrobial, antiviral, antitussive, antimutagenic, and antinociceptive effects [93,94]. Moreover, insecticide and allelopathic properties have been pointed out. Interestingly, important commercial applications are known, namely, a Canadian camphor-based drug (714-X) to treat breast and prostate cancer [95]; a herbal preparation, Padma 28, effective against chronic inflammatory diseases [96]; and preparations in the form of creams, balms, or oils to reduce inflammation and pain in muscles.

Eucalyptol or 1,8-cineole is also always present in species of the section *Stoechas*, mainly in *L. viridis*. This compound is a monoterpenoid oxide and has been mostly extracted from the Lamiaceae, Zingiberaceae, and Myrtaceae families, namely, from *Eucalyptus* sp. essential oil, which contains high concentrations (above 90%) of this compound. Moreover, 1,8-cineole can be available in a standardized type for clinical uses [97]. Although 1,8-cineole is broadly found in essential oils, the content of 1,8-cineole from plants varies with ecology, environment, and other factors. On the other hand, regarding industrialized extraction and isolation of 1,8-cineole, the standardization of plant production with sufficient essential oil content, and the high cost of isolation have become inconvenient tools. To overcome these limitations, synthetic molecules were produced, by using heteropoly acids as the solid acid catalyst for the isomerization of α-terpineol to 1,8-cineole.

Another way to achieve chemical compound synthesis is the development of microbial metabolic engineering strategies. Owing to its pleasant aroma, 1,8-cineole is often used as a flavoring agent in cosmetics, fragrance, and food products, and as an insect repellant. 1,8-Cineole is frequently reported for the treatment of respiratory diseases applied in drug formulations as a decongestant and antitussive stimulator, being the anti-inflammatory, antioxidant, anticancer, antimicrobial, antiviral, sedative, and analgesic effects of some of the main pharmacological properties [98]. For example, its antiviral activity was reported in some studies to protect against influenza viral infections [97]. Moreover, a recent in silico study revealed the potential of 1,8-cineole to inhibit the main protease (Mpro) of SARS-CoV-2 [99].

Fenchone is also present at considerable concentrations in *Lavandula* section *Stoechas* species, except in *L. viridis* essential oils. It is one of the main compounds reported in *L. pedunculata* and *L. stoechas* subsp. *stoechas*. The most important members of the fenchane group of bicyclic monoterpenes are the secondary alcohol fenchol and the corresponding ketone fenchone. Fenchone is a substituted norbornane with a ketone functional group and three additional methyl groups and is derived via the rearrangement of a bicyclic precursor structurally. Norbornanes are known for their strained bonds and angles, as they are constrained to have the cyclohexane ring in an envelope configuration, which gives rise to high reactivity [100]. Fenchone has a structure and odor like camphor, derived from the bicyclic precursors, either from geranyl diphosphate or neryl pyrophosphate. This pathway depicts the cyclization of geranyl diphosphate to form (-)-endo-fenchol, followed by the rearrangement of (-)-endo-fenchol to α-fenchene. A dehydrogenation step then changes α-fenchene to α-fenchocamphorone. Fenchone is mainly used in perfumery and as a food flavoring agent.

### 3.2. Non-Volatile Compounds

In comparison to the large number of studies that characterize *Lavandula* essential oils, the non-volatile fraction (e.g., phenolic compounds and high molecular weight terpenes) remains poorly described, with only a few studies reporting their chemical composition. Moreover, some studies are often qualitative, reporting only the presence or absence of phenolic compounds, and the equipment used for analytical identification and quantification is not always the same, thus compromising comparisons between reported chemical contents. Indeed, in Table 2, the absence of compounds in some species may not reflect a real absence, but the lack of studies on this species. Generally, these types of compounds are present in polar extracts, the maceration of raw material into a solvent is usually the extraction method mostly used for phenolic compound extraction. The solvents most reported are water, ethanol, methanol, acetone, or their mixtures with water, and more non-polar solvents such as chloroform, ethyl acetate, diethyl ether, and hexane. However, there are many techniques to extract non-volatile compounds from plants with microwave-assisted, ultrasounds, or Soxhlet apparatus being some of the most employed [8,25,48]. In addition, analytical factors, such as the extraction solvent used, strongly influence the concentrations and the type of chemical compounds isolated. The extraction of phenolic compounds is influenced by the nature and the polarity of the solvent, temperature, and extraction time. Indeed, the presence of diverse phenolic compounds with different chemical characteristics and polarities may compromise their solubility in the selected solvent [101]. Among the non-volatile secondary metabolites isolated from plants, phenolic compounds are the most studied, being characterized by the presence of at least one aromatic ring with one or more hydroxyl groups connected [102]. According to the number of phenolic rings and the structural elements that bind rings to one another, these compounds can be classified as simple phenols, phenolic acids, flavonoids, xanthones, stilbenes, and lignans. These compounds are synthesized during plant growth and/or due to stress conditions such as infection, cutting, or exposure to UV radiation [103,104].

In plants, phenolic compounds can be present in their free form and/or linked to sugars or proteins. In recent years, the biological activity of these compounds has been reported, namely, their antioxidant, antimicrobial, anti-inflammatory, and insecticidal effects, and others. Phenolic compounds are widely used in the food industry as a natural preservative (antioxidant and antimicrobial agents), as a natural pigment, or as a functional additive [111]. In the pharmaceutical industry, these compounds are also extensively employed as therapeutic agents against diabetes, cancer, and cardiovascular and neurodegenerative diseases. Furthermore, in other industries such as cosmetics, packaging, and textiles, phenolic compounds are applied due to their antioxidant, antimicrobial, UV-protective, and pigmentation properties [112]. Regarding *Lavandula* species from section *Stoechas*, phenolic acids, and flavonoids are the most reported phenolic compounds. Furthermore, other non-volatile metabolites such as triterpenes can be found, as detailed in the following sections, and shown in Table 2.

#### 3.2.1. Phenolic Acids

Phenolic acids are a diverse class of the main phenolic compounds produced in plants via the shikimate pathway, through L-phenylalanine or L-tyrosine as the precursor agents [113]. These compounds are generally characterized by hydroxylated aromatic rings and are present in connection with glycosides, amides, or esters, and rarely in their free form [114]. Phenolic acids can be divided into two groups, namely, hydroxybenzoic and hydroxycinnamic acids, which are derived from non-phenolic molecules. The hydroxybenzoic acids (C6-C1 skeleton) are derived from benzoic acid and are found conjugated with sugars or organic acids, whereas the hydroxycinnamic acids (C6-C3 skeleton) are derived from cinnamic acid and are produced as simple esters with glucose or hydroxycarboxylic acids [115].

In *Lavandula* species, some studies that compare the aqueous and hydroethanolic/ethanolic extracts report that the aqueous extract has a higher concentration of total phenolic compounds than the more non-polar ones [6,8]. Phenolic acids represent a significant content of the phenolic composition in these species, particularly in those from section *Stoechas* as pointed out in Table 2.

For example, rosmarinic acid, which is the main compound present in all compiled species, has a maximum value of 550 mg/g in *L. pedunculata* aqueous extracts, 73 mg/g in subsp. *luisieri*, 74 mg/g in subsp. *stoechas*, and 38.8 mg/g in *L.viridis* hydroethanolic extracts [8,25,108,109]. The presence of this caffeic acid dimer is characteristic of the family Lamiaceae and is also responsible for its potential biological activities [114,116,117]. Other compounds bellowing to this class of compounds, such as salvianolic acid B (582 mg/g) and lithospermic acid A (26 mg/g), were also reported in *L. pedunculata* aqueous extract and chlorogenic acid (18.5 mg/g) in *L. stoechas* subsp. *stoechas* methanolic extract [8,25,108]. Salvionic acid B, a rosmarinic acid dimer, was reported as having the highest content of hydroxycinnamic acid in extracts of the *Stoechas* section. The occurrence of this type of caffeic acid tetramer, mainly present in the genus *Salvia*, was reported for the first time in *L. stoechas* by Algieri et al. [106]. Other caffeic acid trimers, such as salvionic acid A and C, lithospermic acid A, and yunnaneic acid F, were also reported in *L. pedunculata* and *L. stoechas* [6,8,106,107].

#### 3.2.2. Flavonoids

Flavonoids are plant pigments, mainly responsible for the color of flowers. They are characterized by a 15-carbon skeleton, arranged as C6-C3-C6, with diverse substitutions, arrangements of the base skeleton, or unsaturation degrees, thus giving rise to different subclasses. Their structures consist of two aromatic rings linked to a 3-carbon bridge, containing a heterocyclic ring [113]. They are classified into flavanones, flavones, isoflavones, flavanols, flavonols, anthocyanidins, and chalcones based on the oxidation level of their heterocyclic ring. The flavonoids are biosynthesized through a combination of shikimic acid and acetate pathways [118]. The basic flavonoid skeleton can have numerous substituents, such as sugars or hydroxyl groups. Sugars are very common with the majority of flavonoids, existing naturally as glycosides. Both sugars and hydroxyl groups increase the water solubility of flavonoids, while other substituents, such as methyl groups and isopentyl units, make flavonoids lipophilic. They are present in high concentrations in the epidermis of leaves and fruit peel and have important and varied roles as secondary metabolites.

In plants, flavonoids are involved in diverse processes such as UV protection, pigmentation, stimulation of nitrogen-fixing nodules, and disease resistance [119]. Flavonoid subclasses observed in the genus *Lavandula* include flavones, flavonols, flavanones, and anthocyanidins. These can occur in a free form (aglycones) or conjugated with *O*- or *C*-glycosides. In the conjugated form, the glycoside moiety is usually a glucoside, glucuronide, or rutinoside. Some flavonoids from the *Lavandula* species, especially anthocyanidins, are also subjected to acylation with malonic and coumaric acids. Regarding flavonoids, Upson and Andrews [1] revealed the presence of these compounds in the seven sections of *Lavandula*. In addition to 7-*O*-monoglycoside flavones, the most common type of flavonoids found in the Lamiaceae family, are di-*O*-glycoside flavones that are present exclusively in the section *Stoechas*, which can be a chemical marker of this section. Contreras et al. also corroborated these findings by showing the absence of the *C*-glycosides and hydroxylated flavones in *L. stoechas*, contrasting with the presence of these types of flavones in *L. dentata* that belong to the section *Dentatae* [107]. Compounds such as luteolin-7-*O*-glucuronide, methylluteolin-*O*-glucuronide, eriodictyol-*O*-glucuronide, and luteolin 7-*O*-glucoside are the most representative in *L. pedunculata*, *L. stoechas* subsp. *stoechas* and *L. viridis* [8,60,106,108,109]. Interestingly, pinocembrin flavanone, present mainly in *Euphorbia*, *Eucalyptus*, or *Pinus* species, was also reported in *L. viridis* hydroethanolic and ethanolic extracts [110,120]. As previously mentioned, only a few studies report the phenolic composition of extracts from the section *Stoechas* and, therefore, the absence of flavonoids in *L. stoechas* subsp. *luisieri* does not necessarily mean that they are not present in these extracts.

#### 3.2.3. Other Non-Volatile Phytochemicals

Low molecular terpenes are mainly found in essential oils, while in most non-polar extracts, high molecular terpenes such as triterpenoids can occur. Triterpenoids are widely distributed in plants, either in the free form (aglycone) or as glycosides, or in other combined forms. Structurally, they are composed of 30 carbon atoms consisting of six isoprene units, and they are biosynthesized by the cyclization of the squalene subproduct of the IPP and DMAPP pathways [121,122]. According to their chemical structure, triterpenoids can be grouped into linear to pentacyclic compounds. The pentacyclic triterpenoids such as tormentic (138.5 mg/g), oleanolic (34.7 mg/g), and ursolic acids (124 mg/g) were identified in *L. stoechas* subsp. *luisieri* ethanolic extract [25]. The last two triterpenoids frequently occur simultaneously due to their similar structural form. All of these pentacyclic triterpenes are associated with great pharmacological potentials, such as antitumor, anti-inflammatory, and antimicrobial effects [123,124]. Due to the low polarity of the ethanol extract, a cadinene-type sesquiterpene derivative, the 3-oxo-cadinol, was also identified [25]. Recently, in *L. stoechas* extract two new copaane sesquiterpenoids, the stoechanones A and B, were identified by spectroscopic techniques (NMR and HRESIMS). These compounds have shown a high herbicidal potential [26]. Moreover, 5-hydroxymethyl-2,3,4,4-tetramethylcyclopent-2-en-1-one (79 mg/g) was also reported in *L. stoechas* subsp. *luisieri* SFE extract. Typically, this type of compound has been also reported in the essential oil of *L. stoechas* subsp. *luisieri* [62,72,75,76].

## 4. Biological Activities

Since ancient times *Lavandula* species have been used in traditional medicine to treat many diseases, which indicates that aromatic and medicinal plants play an important role in human health, due to their biological properties. In the literature reviewed, terpenes, phenolic acids, and flavonoids are always present as the main chemical constituents in *Lavandula* extracts including volatile, polar, and non-polar extracts. These phytochemical substances are responsible for most of the reported biological activities. Essential oils and non-volatile extracts of species from the section *Stoechas* have been studied by several authors and their biological effects have shown promising potential to be used in pharmaceutical, cosmetic, or food applications. Properties such as antioxidant, antimicrobial, anti-inflammatory, antitumor, immune system protector, insecticide, and other effects have been described to these species, as systematized next.

### 4.1. Antioxidant Activity

Recently, the interest in the role and use of natural antioxidants has increased as a strategy to prevent oxidative damage in several health disorders, in which oxidative stress plays a relevant role. Regarding the assessment of free radicals, oxidative stress, and antioxidant activity, although several methods have been developed, no single method is truly adequate for estimating the total antioxidant capacity of the extracts due to different mechanisms of action. Indeed, according to the mechanism of action, hydrogen atom transfer and single electron transfer techniques are generally used. The first technique measures the capacity of an antioxidant to catch free radicals by hydrogen donation and includes the oxygen radical absorbance capacity (ORAC), total radical-trapping antioxidant (TRAP), thiobarbituric acid reactive substances (TBARS), and chemiluminescence (CL) methods. The second mechanism is based on the electron transfer reduction capacity of an antioxidant compound, such as ferric antioxidant power (FRAP) or cupric reducing antioxidant capacity (CUPRAC). Additional methods, such as the DPPH (2,2-diphenyl-1-picryl-hydrazyl-hydrate) and Trolox equivalent antioxidant capacity (TEAC), are based on both hydrogen and single electron transfer. In these cases, the radicals can be scavenged by electron reduction or radical quenching that involves hydrogen transfer. Generally, these methods are applied in vitro, while the lipid and glutathione peroxidase and catalase activity assays (the enzymatic antioxidant methodology) are used in vivo [125]. Phenolic compounds appear to be the main agents responsible for the antioxidant activity of polar extracts. They are very effective eliminators of most oxidized molecules, acting as free radical scavengers or metal chelators [126].

The antioxidant activities of *Lavandula* have been addressed using several model systems, such as DPPH, TEAC, ORAC, FRAP, and CUPRAC. Table 3 summarizes the antioxidant activity of the essential oils and non-volatile extracts reported for all species of section *Stoechas*. Considering the importance of phenolic compounds, it is relevant to correlate their content with the antioxidant potential of each plant extract. Indeed, several studies have reported a positive correlation between the phenolic and flavonoid content and antioxidant potential in these species [7,127,128]. Some studies compared this property and compared different species as well as essential oils and different polarity extracts. Baptista et al. revealed that the essential oil, as well as the non-polar and methanolic extracts of *L. pedunculata* and *L. stoechas* subsp. *luisieri,* showed high antioxidant activity (greater for the last species) [128].

As expected, essential oils showed the lowest values for phenolic and flavonoid contents in comparison to methanolic and water extracts, thus revealing that terpenes also have some antioxidant power, mainly responsible for the antioxidant activity of essential oils. Carrasco et al. confirmed that linalool and thymol are responsible for the antioxidant activity of *L. stoechas* essential oil [55]. Similar results were observed by Pereira et al., who reported a considerably higher level of phenolic and flavonoid content in *L. stoechas* subsp. *luisieri* than *L. pedunculata* [7]. Caffeic acid and rutin, phenolic acid, and a flavonoid, respectively, also demonstrated antioxidant effects [133]. The ranking of *Lavandula* spp. essential oils with greater antioxidant power was reported by Matos et al., who revealed that *L. stoechas* subsp. *luisieri* essential oil was the most active, followed by *L. viridis* and lastly *L. pedunculata* [5].

### 4.2. Antimicrobial Activity

Some methodologies have been developed to evaluate the antimicrobial activity of plant products, with diffusion methods widely applied. For example, the agar disk-diffusion assay which is the official method for antimicrobial susceptibility tests was accepted and approved for publication in the Clinical and Laboratory Standards Institute (CLSI). Nevertheless, other diffusion methods are also widely used such as the agar well diffusion, agar plug diffusion, the antimicrobial gradient method, or the Etest commercial version (BioMérieux, Marcy-l’Etoile, France). Likewise, dilution methods are frequently reported for the determination of the minimum inhibitory concentration (MIC). Both agar or broth dilution assays are used, being the micro- or macro-dilution the most used for antimicrobial susceptibility testing [134].

Terpenes and phenolic compounds present in essential oils and non-volatile extracts of *Lavandula* species have also been reported due to their antimicrobial action. The great effectiveness of essential oils does not seem to be related only to the presence of their major compounds, but many times to the synergistic effect between several components. Due to the complex mixture of essential oils, they do not act on specific targets in the cells, so no adaptation or resistance to the essential oils has been reported [135,136]. Some studies have shown the antimicrobial activity of *Lavandula* essential oils and also that of their major compounds, such as linalool, camphor, and 1,8-cineole. Importantly, these results corroborate that the activity of the volatile extracts is not due to just one constituent [38,82,85]. From the southwest and center of Portugal, the antimicrobial activity of two native species, *L. pedunculata* and *L. stoechas* subsp. *luisieri,* was evaluated for the same microorganisms. Both species showed high antimicrobial effects, but the latter species was more effective, presenting lower MIC values [7,128].

Concerning the effect of extracts, the solvent used in the extraction process seems to influence the antimicrobial activity observed, being the non-polar extracts and essential oils, in general, more active than the polar extracts [8,21,128]. The results from the literature on the antimicrobial activity of species from the section *Stoechas* are presented in Table 4. This activity was evaluated in several microorganisms including Gram-negative (16) and Gram-positive (15) bacteria, yeasts (14), and numerous filamentous fungi (31). Overall, Gram-negative bacteria were reported as the most resistant to the action of several antibacterial agents, due to differences in cell structure. Gram-negative bacteria have a strong layer of lipoprotein and lipopolysaccharides, which restricts the diffusion of hydrophobic compounds through the cell [44,47,137]. Most of the reported bacteria are pathogenic species, such as *Escherichia coli*, *Klebsiella pneumonia*, *Salmonella enterica*, *Haemophilus influenzae*, and *Pseudomonas aeruginosa*, with the essential oils and extracts of *Lavandula* species of section *Stoechas* inhibiting their growth. In addition, Gram-positive bacteria such as *Bacillus cereus, Listeria monocytogenes*, and *Staphylococcus aureus* were susceptible to essential oils and extracts. The *Lavandula* extracts revealed inhibitory action against *Candida* spp., some dermatophytes strains, *Aspergillus* spp., food spoilage species (*Rhizopus stolonifer* and *Penicillium* spp.), and some pathogenic fungi of plants (*Alternaria alternaria* and *Fusarium oxysporum*).

Interestingly results against the protozoa *Leishmania* spp. have also been reported. The antileishmanial activity of *L. stoechas* essential oil was evaluated against *Leishmania infantum, L. tropica*, and *L. major*. The concentration of *L. stoechas* essential oil that reduced 50% of the parasite cell (IC_50_) was found for *L. major* (0.9 µg/mL) and *L. infantum* (7 µg/mL). However, against *L. tropia,* the inhibition effect was not found in concentrations up to 10 µg/mL, and these results showed more activity than the positive control (glucantime) [57]. Moreover, Essid et al. reported a significant inhibitory effect of *L. stoechas* essential oil against *Leishmania* spp. [143].

### 4.3. Anti-Inflammatory and Analgesic Activities

Inflammation is recognized as a biological process in response to tissue injury. There are several methods for determining the anti-inflammatory activity of plant extracts or their constituents, which vary according to what is intended to be inhibited at the level of the inflammatory cascade. In vitro studies, mainly aim to assess the inhibition of the activity of enzymes and/or mediators of inflammation, such as nitric oxide (NO), arachidonate 5-lipoxygenase (5-LOX), human leukocyte elastase, and interleukin-6, and elucidate the mechanisms of action involved. Normally the evaluation of the inhibition of inflammatory enzymes or mediators is carried out spectrophotometrically or by molecular biology methods, such as Western blot analysis [144,145,146]. Many studies also assess the anti-inflammatory activity using in vivo systems, where some of the above methodologies are assessed in rats or mice. Usually, the carrageenan-induced edema model is used to assess the contribution of plant extracts/compounds in resisting the biochemical changes associated with acute inflammation [147]. The anti-inflammatory activity potential of essential oils and extracts from some species of the section *Stoechas* has been reported. The essential oil of *L. stoechas*, rich in 1,8-cineole, showed an anti-inflammatory effect able to reduce carrageenan-induced paw edema with an effect similar to that of the positive control (indomethacin). Dermal application of the oil, at doses of 82 and 410 mg/kg, considerably reduced the acute paw edema in mice [61]. As has been reported with other biological activities, the anti-inflammatory effects seem to be due to the complex mixture of chemical compounds, and not to a single component. As already mentioned, the essential oil of *L. stoechas* is mainly composed of fenchone, camphor, and 1,8-cineole, of which the first two revealed a slight inhibition of lipoxygenase (LOX), which can contribute to the moderate anti-inflammatory activity revealed by the essential oil [55]. *L. stoechas* subsp. *luisieri* essential oil (200 mg/kg) showed anti-inflammatory activity through the inhibition of carrageenan-induced rat paw edema (83%) and analgesic effects (67%), with a stronger action compared to the positive control [20].

The potential use of *L. stoechas* as an herbal remedy for gastrointestinal disorders is justified by the results of its methanolic extracts, rich in rosmarinic acid, which showed a potent anti-inflammatory action, presenting an intestinal anti-inflammatory effect in a murine epithelial cell line (CMT-93) [148]. In comparison with *L. dentata* extract, only *L. stoechas* extract exhibited an anti-inflammatory effect by appreciably inhibiting carrageenan-induced paw edema in mice [106,107]. In addition, alcoholic extracts of *L. stoechas* inhibited the inflammation induced by carrageenan in rats [131,149]. Their ethanolic (at 2000 mg/kg) and hydroethanolic extract produced a significant inhibition of inflammation (74%) compared to 1% diclofenac [131]. Flavonoid and mucilage extracts of *L. stoechas* also showed a significant reduction in edema of about 85% and 62%, respectively, demonstrating that these compounds in *L. stoechas* extracts seem to be responsible for the anti-inflammatory activity observed [150]. Moreover, the ethyl acetate extracts of *L. stoechas* showed inhibition of the lipopolysaccharide (LPS)-induced inflammation in RAW 264.7 macrophages [151]. In another experimental model, *L. stoechas* methanolic extract revealed anti-inflammatory activity in vivo (10 and 25 mg/kg), with values comparable to those registered for glucocorticoid dexamethasone, a steroidal anti-inflammatory drug. This anti-inflammatory effect was explained by the regulation of inflammatory precursors, such as inducible nitric oxide synthase, pro-inflammatory cytokines, and cyclooxygenase 2 [106]. Significant results were reported in *L. pedunculata* extracts through an in vitro model of lipopolysaccharide (LPS)-stimulated macrophages, showing the inhibition of nitric oxide (NO) production, an important inflammatory marker [8]. These authors compared the anti-inflammatory potential through IC_50_ (the extract concentration corresponding to 50% of inhibition of the NO production in comparison with the negative control, 100% of NO production) between aqueous and hydroethanolic extracts, which revealed values of IC_50_ of 140 µg/mL for aqueous extract and 124 µg/mL for hydroethanolic extract. Zuzarte et al. also reported the inhibition of NO production by *L. stoechas* essential oil at 0.16 to 0.32 µL/mL, without affecting cell viability [62]. For *L. stoechas* subsp. *luisieri*, at non-cytotoxic concentrations (up to 200 µg/mL), the essential oil showed significant inhibition of inflammatory markers (iNOS expression and NF-ƙB activation) in intestinal C2BBe1 and human chondrocytes [74].

### 4.4. Anti-BACE-1 and Anticholinesterases Activities

The usual aspects of neurodegenerative diseases include loss of cholinergic neurons and an increase in the activity of cholinesterase, butyrylcholinesterase (BChE), and acetylcholinesterase (AChE). These enzymes are responsible for the rapid hydrolysis of acetylcholine in cholinergic synapses [152]. Another pathological characteristic of neurodegenerative diseases, such as Alzheimer’s disease, is the accumulation of amyloid plaques (produced by β- and γ-secretase) [153]. Thus, the new treatments for neurodegenerative disorders include the inhibition of these types of enzymes.

Medicinal plants have been widely used as memory enhancers and in dementia therapy, mainly as β-secretase (BACE-1), AChE, and BChE inhibitors. Due to the low molecular weight and high hydrophobicity of terpenoids, they have a good chance of crossing cell membranes and the blood–brain barrier. The essential oil and necrodane components of *L. stoechas* subsp. *luisieri* were reported as showing a promising effect on the treatment of Alzheimer’s disease. The essential oil (90 µg/mL) and, namely, 2,3,4,4-tetramethyl-5-methylene-cyclopent-2-enone (45 µg/mL) inhibited BACE-1 activity by enzymatic and cellular assays [73]. Concerning cholinesterase inhibition, *L. stoechas* methanolic extract showed appreciably reduced levels of AChE and malondialdehyde in the brain of mice. Further, it was demonstrated that *L. stoechas* can be useful in attenuating the dementia process, reducing the oxidative process of neurons, and decreasing the neurodegradation of cholinergic transmission in the brain of mice [134]. *L. pedunculata* polar extracts and essential oil (at a concentration of 2.5 mg/mL) were active against AChE and BChE, with the essential oil revealing the most effective inhibitor action for AChE (57%) compared to polar extracts (45%); however, for BChE, no significant differences were observed between the essential oil and non-volatile extracts [6]. Ferreira et al. also revealed a higher anticholinesterase (AChE) activity for *L. pedunculata* essential oil compared with the ethanolic extract, thus suggesting the potential of terpenes in the inhibition of cholinesterases [129]. Furthermore, *L. viridis* methanolic extract showed a strong inhibitory effect of AChE and BChE activities in vitro and in vivo [109]. *L. viridis* essential oil and supercritical fluid extract revealed AChE inhibition, with the essential oil being the most effective AChE inhibitor, while the supercritical extract was the most effective BChE inhibitor. The inhibition of AChE and BChE was evaluated for the main compounds of the *L. viridis* essential oil, with 1,8-cineole being the most active compound [30]. Studies on cholinesterase inhibition (AChE and BChE) after the digestion process were reported by Costa et al., which showed that only the inhibition of BChE was affected after in vitro gastric and pancreatic digestions [154].

### 4.5. Cytotoxic Activity

Cytotoxicity assays are extensively used in in vitro cytotoxic studies and are generally performed against normal and tumor cell lines. The methodologies mostly applied to quantify cell viability resort to 3-(4,5-dimethyl-2-thiazolyl)-2,5-diphenyltetrazolium bromide (MTT), Alamar blue, or neutral red (NR), through spectrophotometric measurement. In addition, a protein method is reported, the lactate dehydrogenase leakage (LDH) assay [155].

The effect of *L. stoechas* subsp. *luisieri* on the viability of normal and cancer cells has been investigated. The hydroethanolic extracts and essential oil of this species did not affect the viability of normal cells, namely, adherent human skin fibroblasts (BJ) and mouse macrophages (RAW264.7) at low concentrations (<3.2 mg/mL and <0.08 µL/mL, respectively) [21,36]. Similarly, no cytotoxic effect was observed in a porcine liver cell line (PLP2) and human adult keratinocytes (HaCat) for the aqueous, hydroethanolic, and methanolic extracts of *L. pedunculata* [7,8]. Further, in *L. stoechas* subsp. *stoechas,* the hydromethanolic extract showed a low cytotoxic effect (up 62.5 µg/mL) on RAW264.7 [142]. Concerning tumor cells, anti-proliferative effects were observed in species of the *Stoechas* section. Lopes et al. reported that *L. pedunculata* extracts were effective in inhibiting the growth of breast adenocarcinoma (MCF-7), lung cancer (NCI-H460), cervical carcinoma (HeLa), and hepatocellular carcinoma (HepG2) [8]. The hydroethanolic and aqueous extracts of *L. stoechas* subsp. *luisieri* also showed cytotoxic effects in hepatocellular carcinoma (HepG2) [21]. Ethyl acetate and n-butanol extracts of *L. stoechas* were also evaluated concerning the cell viability of macrophages (RAW264.7), preadipocytes (3T3L1), and rat H4IIE hepatoma. It was observed that both extracts did not affect the viability of the cells (up to 200 µg/mL for the first type of cells and up to 50 µg/mL for the last two) [151]. The same authors verified cytotoxic effects on the myotubes cell line (C2C12) of both *L. stoechas* extracts. In the RAW 264.7 cell line, the viability was not affected by *L. stoechas* essential oil at concentrations up to 0.32 µL/mL, which seems to reveal a safer potential of the essential oil compared to polar extracts [62]. Essential oils of *L. stoechas* and *L. stoechas* subsp. *stoechas* were highly effective in cancer cells, showing very low IC_50_ values (0.035–18 µg/mL), in human gastric adenocarcinoma (AGS), melanoma (MV3), breast carcinoma (MDA-MB-231) for *L. stoechas*, and in human colon cancer (COL-2) and hormone-dependent human prostate cancer (LNCaP) for *L. stoechas* subsp. *stoechas*, thus confirming a potent in vitro antitumor effect of these volatile extracts [61,80]. Beyond the essential oil, Gören et al. also revealed a cytotoxic effect of *L. stoechas* subsp. *stoechas* chloroform extract against P-388 (mouse leukemia) cells [80]. Moreover, *L. viridis* methanolic and hydroethanolic extracts (500 µg/mL) and their main compound, rosmarinic acid (125 and 250 µg/mL), did not show any toxic effect on colon adenocarcinoma (CaCo-2) cell viability [154].

**Figure 3:**
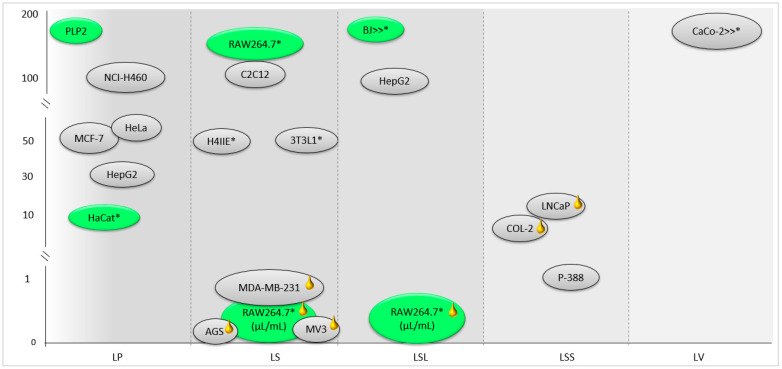
Screening assays on the cytotoxic activity of essential oils (yellow mark) and extracts of *Lavandula* sp. section *Stoechas* in normal (green) and tumor (grey) cells. Cells with * correspond to the cell viability concentration values. LP: *L. pedunculata* [7,8]; LS: *L. stoechas* [61,62,151]; LSL: *L. stoechas* subsp. *luisieri* [21,36]; LSS: *L. stoechas* subsp. *stoechas* [80]; LV: *L. viridis* [154].

A summary of the cytotoxic effects in non-tumor and tumor cells of the essential oils and extracts of the *Lavandula* sp. section *Stoechas* can be observed in Figure 3. Some studies have reported that the cytotoxic activity of essential oils and other extracts is selective, as the anti-proliferative effect in tumor cells was stronger in comparison to normal cells, thus suggesting that these extracts are promising agents for anticancer treatments.

### 4.6. Other Pharmaceutical Activities and Bioavailability

For *Lavandula* section *Stoechas,* other biological activities, namely, the anti-hyperglycemic, anticonvulsant, hepatic, renoprotective, reproprotective, and spasmolytic effects were also reported. The anti-hyperglycemic potential of *L. stoechas* hydroethanolic extract and essential oil in alloxan-induced diabetic mice was demonstrated by significant effects on the reduction in blood glucose levels compared to pioglitazone, the reference drug [22,53]. The essential oil of *L. stoechas* showed potential hepatic, renoprotective, and reproprotective effects against malathion-induced oxidative stress injury in the liver and kidney, and steroidogenesis disruptions in mice. This beneficial effect of the essential oil appears to be related to its antioxidant properties and ability to eliminate free radicals [23,59]. It was also reported that the hydromethanolic extract of *L. stoechas* (600 mg/kg) possesses an anticonvulsant activity, reducing the severity of convulsions and lethality induced by pentylenetetrazole (PTZ) in mice. At this concentration, although no hypnotic effect in mice was observed, the animals were considered calm and relaxed, revealing a sedative effect. A spasmolytic effect of hydromethanolic extract has also been reported in rabbits at concentrations of 0.1–1 mg/mL, causing inhibition of spontaneous fasting contractions. According to the authors, these activities seem to be mediated by the same mechanism, namely, through the calcium channel blockade [156]. These beneficial properties are generally assessed using in vitro assays; nevertheless, to translate these findings to the clinic, the bioavailability of these extracts/compounds in the organism must be considered. Importantly, bioavailability is influenced by the digestion and metabolization process, where the chemical compounds can be subject to several chemical changes, such as biotransformation, thus compromising their availability and biological properties in the organism. Although numerous studies report high phenolic composition and biological activities in this section, only a few studies have focused on bioavailability. According to Celep et al., who evaluated the effect of simulated digestion in *L. stoechas* subsp. *stoechas* methanolic extract, the phenolic and flavonoid content was not affected by the digestion process. Concerning the major compounds, chlorogenic acid showed better bioavailability when compared to flavone glycosides. Regarding the antioxidant activity, a slight reduction was observed after digestion [108]. Costa et al. also reported a significant reducing effect on the concentration of rosmarinic acid in *L. viridis* extracts by gastric fluid contrasting with the pancreatic fluid. This work showed that the antioxidant activity of the methanol extract and rosmarinic acid was maintained after in vitro gastrointestinal processes [154].

### 4.7. Insecticidal, Nematicidal, and Ixodicidal Activities

The use of chemical insecticides is presently the most popular practice for controlling insects. However, the exhaustive use of these chemical products is responsible for the emergence of resistant insects, has negative impacts on the environment, and is involved in food chain contamination. Thus, the search for new alternatives to avoid such damage is imperative, and the use of molecules with natural origins, such as essential oils, seems to be a promising alternative to control insects and larvae. The insecticidal activity varies with the concentration of essential oil used, species of insects tested, and time of exposure [157,158]. Bachiri et al. evaluated the insecticidal effect of *L. stoechas* and *L. pedunculata* essential oils and showed high activity against the wheat pest *Tribolium castaneum*, causing the mortality of the individuals after 2 days of treatment. In this study, *L. stoechas* essential oil showed more marked insecticidal effects [159]. Similar effects were observed when the repellent activity was assessed, with 50 µL of the essential oil causing 73% and 93% of repellency, for *L. pedunculata* and *L. stoechas*, respectively. The essential oil of *L. stoechas* showed a high insecticidal effect with low lethal concentrations (LC_50_) for cigarette beetle *Lasioderma serricorne* (3.84 μL/L), lesser grain borer *Rhyzopertha dominica* (5.66 μL/L), and *Tribolium castaneum* (39.69 μL/L) [49]. *L. stoechas* essential oil showed active effects against *Anopheles labranchiae* (a vector of malaria transmission) with LC_50_ of 112.5 mg/L [67]. Furthermore, good insecticidal activity against *Sitophilus granarius* and *S. oryzae* was revealed for *L. stoechas* essential oil [69]. For *L. stoechas* subsp. *luisieri*, González-Coloma et al. reported antifeedant effects caused by the main compounds (*trans*- and *cis*-α-necrodyl acetate, and fenchone) and by the essential oil against *Spodoptera littoralis*, *Leptinotarsa decemlineata*, and *Myzus persicae*. Only *M. persicae* was considerably affected by the action of these two chemical compounds, while the essential oil also revealed a feeding inhibition in *S. littoralis* and *L. decemlineata*. According to these results, antifeedant properties cannot be attributed to the major compounds individually, which, once again, supports the existence of synergistic effects among the essential oil components [71]. Afterward, González-Coloma et al. verified the antifeedant effects of *L. stoechas* subsp. *luisieri* essential oil and a methanolic extract, from wild and cultivated plants, against *Rhopalosiphum padi*, *S. littoralis*, and *M. persicae*. As in the previous study, a significant antifeedant effect was reported for the essential oils and methanolic extract, but the latter was less effective. Moreover, the ethanolic extract was inactive against *M. persicae*; however, a supercritical fluid extract with a high concentration of necrodane-type ketones showed strong *S. littoralis* antifeedant effects [72]. Some of the major components of *L. stoechas* subsp. *luisieri*, such as 2,3,4,4-tetramethyl-5-methylidenecyclopent-2-en-1-one, hydroxymethyl-2,3,4,4-tetramethylcyclopent-2-en-1-one, and 3-oxo-cadinol, showed antifeedant effects against *S. littoralis* [25].

Beyond the antifeedant activity, also, nematicidal effects were reported for the necrodane derivatives against *Meloydogine javanica*, in contrast to the aqueous extract [160]. Julio et al. presented a new class of ixodicidal agents from the essential oil and hexane extract and organic fraction of the residual hydrolat of *L. stoechas* subsp. *luisieri* against *Hyalomma lusitanicum* [161]. This promising activity was attributed mainly to the presence of necrodane derivatives, namely, 3,3,4,5-tetramethyl-2H-pyran-2,6(3H)-dione and 2,2,3,4-tetramethyl-5-oxocyclopent-3-en-1-yl-methyl acetate.

### 4.8. Phytotoxicity and Allelopathic Activity

Phytotoxic effects were reported for the necrodane derivative compounds of *L. stoechas* subsp. *luisieri* against *Lactuca sativa* and *Lolium perenne*, with the latter showing more sensibility [160]. Masi et al. reported that stoechanones A and B isolated from organic extracts of *L. stoechas* showed phytotoxic effects against seed germination and seedling growth of *Amaranthus retroflexus*, with a strong inhibition in the seed germination and radical and hypocotyl lengths of seedlings, suggesting a potential herbicide activity [26]. On the other hand, Hassiotis and Orfanoudakis studied the effect of leaves–flowers and the essential oil of *L. stoechas* on the development of two mycorrhizal species (*Septoglomus deserticola* and *Rhizophagus intraradices*) in a host plant, *Allium porrum*. It was verified that a small number of leaves–flowers (up to 2 g/L of soil) or essential oil (up to 25 mg/L of soil) were beneficial for fungi infection and the effects of allelopathy were also confirmed; however, it was revealed that higher concentrations induced the inhibition of these mycorrhizal species [68].

### 4.9. Phytostabilisation of Soils

Phytostabilization involves the reduction in the mobility of heavy metals in soils. Populations of *L. pedunculata* growing in soils with multi-elemental contamination under Mediterranean conditions were studied by Santos et al. Their phytostabilization potential was confirmed by the accumulation of elements in roots, avoiding their translocation to the aerial parts, and no phytotoxic effects on plants were observed [162]. Similar behavior has also been reported in the growth of *L. stoechas* in mercury-rich soil, in which it was observed that the roots act as a barrier, revealing very low concentrations of mercury in the leaves that could contribute to the phyto-immobilization of this element in the soil [163].

## 5. Conclusions

**Figure 4:**
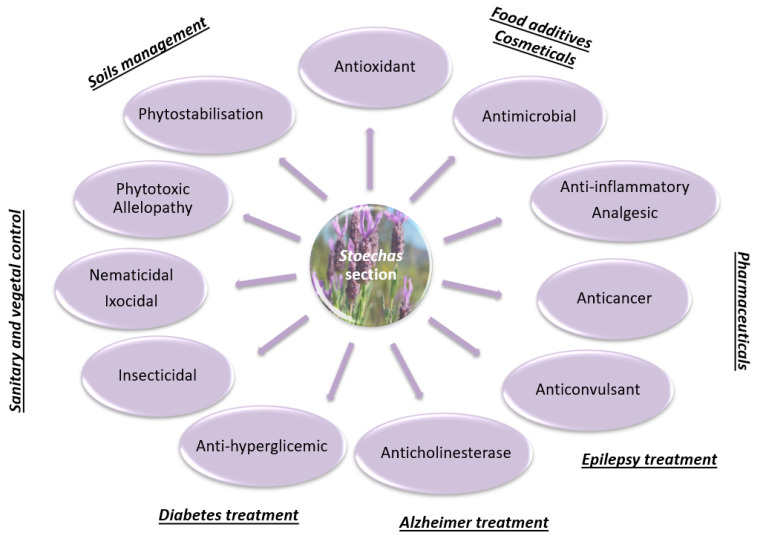
Flowchart representation of the biological activities of *Lavandula* section *Stoechas* and their potential applications.

The present review focused on the phytochemical characterization and biological properties of the section *Stoechas* mainly present in Mediterranean areas. The remarkable diversity of chemical compounds found in these species is systematized for the first time. This qualitative and quantitative chemical diversity is mainly due to genetic differences and diverse environmental conditions that influence the plant’s biological properties. Therefore, a proper chemical characterization is imperative to distinguish different species, which are sometimes very similar morphologically. Indeed, due to the similar morphology, these species are sometimes mistaken and undifferentiated, which can lead to incorrect labeling and, consequent, misuse. Bearing in mind that this difficulty does not arise only in these species, and given the insufficient regulation and control by official organisms, we believe that studies on plant phytochemistry and pharmacology are extremely important and useful for future decisions and the definition of control standards. The biological properties of these plants can be applied in several areas, from pharmaceutical to food additives, cosmetics, and hygienic and vegetal sanitary industries (Figure 4). It was also demonstrated in this review that the species of the section *Stoechas* has a broad spectrum of antimicrobial activity, acting on several classes of microorganisms. Furthermore, the anticancer activity of essential oils and other non-volatile extracts in several cancer lines points out promising opportunities in the clinic. Thus, these extracts/compounds can be considered as an alternative and economical source of natural products that can be employed in numerous industries. Moreover, the knowledge of phytochemistry will also contribute to the cultivation of more valuable species, a sustainable way to increase the progress in less-favored areas, promoting species preservation and, consequently, biodiversity.

## Figures and Tables

**Table 1:** Volatile compounds present in *Lavandula* sp. section *Stoechas* essential oils.

	RI Lit *	*L. pedunculata* ^LP^	*L. stoechas* ^LS^	*L. stoechas* subsp. *luisieri* ^LSL^	*L. stoechas* subsp. *stoechas* ^LSS^	*L. viridis* ^LV^
**Monoterpene hydrocarbons**						
Tricyclene	923	0.3–0.7	0.1–0.7	-	0.1–0.2	0.1–0.7
α-Pinene	936	0.1–10.7	0.06–23.2	0.2–4.3	0.2–6.1	0.3–9
α -Fenchene	949	-	-	-	1.3–2.2	-
Camphene	950	0.7–7.1	0.1–11.4	0.1–0.8	0.2–2.8	0.1–7.7
Sabinene	973	0.1–0.6	0.02–0.1	0.1–0.2	0.1–0.3	0.2–0.3
β-Pinene	978	0.1–9	0.03–13.8	0.1–4.5	0.1–3.2	0.1–1.2
Myrcene	989	0.1–0.4	0.1–0.5	0.1–0.2	0.2–1.7	0.1
δ-3-Carene	1003	4.1	0.1	0.1–1.8	0.3	-
*p*-Cymene	1024	0.2–0.5	0.1–6.5	0.1–4.5	0.02–1.4	0.3–0.5
Limonene	1030	0.8–1.5	0.03–2.7	0.1–0.8	0.04–1.3	0.1
*cis*-β-Ocimene/ *trans*- β-Ocimene	1037/ 1048	0.1–0.8	0.1–2.6	0.1–1.0	-	0.2
**Oxygenated monoterpenes**						
*cis*-α-Necrodol	-	-	-	1.3–3.3	-	-
1,8-Cineole	1031	0.8–34.3	0.04–61.4	1.3–20.6	0.2–17.8	21.3–74
*cis*-Linalool oxide	1075	0.3–0.8	0.1–1.1	0.3–1.6	0.03–0.05	0.4–1.3
Fenchone	1088	0.6–48.7	0.03–68	0.1–22	21–71	-
Linalool	1099	0.5–5.2	0.3–49.9	0.2–6.2	0.02–0.4	1.8–3.8
Thujone	1114	-	8.9–32.1	-	-	-
α -Fenchol	1115	-	2.8	-	-	-
α-Campholenal	1124	0.1–0.4	0.4	0.1–0.2	0.02–0.1	0.4–0.8
*trans*-α-Necrodol	1130	-	-	1.3–13	-	-
*trans*-Pinocarveol	1140	-	1.6–2.1	0.2–1.02	0.01–0.2	0.6
Camphor	1143	3.6–53.1	0.04–71.9	1.1–74.4	7–46.7	2.9–31.6
Menthone	1150	-	9.0	-	12.6	-
Borneol	1166	0.3–3.4	0.4–4.9	-	0.2–1.7	4.6
*p*-Mentha-1,5-dien-8-ol	1167	-	0.3–5.0	0.2–2.3	1.2	-
Lavandulol	1168	-	0.2–3.7	0.3–11.7	-	-
*trans*-linalool oxide	1171	0.2–0.6	0.01–0.9	0.1–3.2	0.1–0.4	0.4–1.8
Menthol	1177	-	-	-	18.1	-
Terpinene-4-ol	1177	0.5–1.7	5.1	0.5	0.5	-
α-Terpineol	1189	0.1–0.9	0.4–13.1	0.1–0.4	0.1–0.5	0.5
Myrtenal	1192	0.2–2.4	0.02–2.4	0.2–0.4	0.02–1.4	0.3–1.2
Myrtenol	1194	-	0.02–2.1	-	0.3–2.8	-
Verbenone	1206	0.2–1.5	0.1–2.7	0.1–2	0.2–0.5	0.3–3.5
Fenchyl acetate	1219	0.1–0.5	3.9	-	0.5–1.1	-
Eucarvone	1222	-	-	1.9	-	-
Pulegone	1234	-	-	-	40.4	-
Linalyl acetate	1253	-	64.3	-	-	-
*trans*-α-Necrodyl acetate	1265	-	-	1.8–48.2	-	-
*cis*-α-Necrodyl acetate	1281	-	-	1.2–5.9	-	-
Bornyl acetate	1283	0.1–3.5	0.08–6.2	0.5–1.6	0.1–6.2	0.2
Lavandulyl acetate	1289	0.1–3.2	0.02–5.7	0.8–7.6	0.2–5.6	-
Thymol	1290	-	0.1–3.1	-	-	-
Carvacrol	1300	1.2	0.02–3.4	-	0.1–0.6	0.2
Myrtenyl acetate	1328	-	1.2–11.7	0.6–2.7	0.6–9.5	0.2
Neryl acetate	1362	-	0.1–1.1	0.6–1.1	-	0.02
Lyratyl acetate	1387	-	-	2.4–3.5	-	-
**Sesquiterpene hydrocarbons**						
α -Gurjunene	1408	-	-	1.4	-	-
Caryophyllene	1420	0.2	0.1–5	0.6	0.04–0.1	-
γ-Selinene	1470	-	2.5	-	-	-
β-Selinene	1486	0.5	0.2–1.4	0.3–12.8	0.1–0.7	0.3
α-Selinene	1493	-	-	1	0.1–0.4	-
γ-Cadinene	1513	0.1–0.9	0.1–5.3	0.7–0.8	-	-
δ-Cadinene	1523	-	0.6–0.7	0.5–1.5	0.8–2.2	-
Selina-3,7(11)-diene	1540	-	-	1.4	-	-
Ledol	1566	-	1.96	0.9–2.9	0.02–0.7	-
Viridiflorol	1590	0.2	0.1–7.4	0.2–12.1	0.9–4.3	0.2
**Oxygenated sesquiterpenes**						
*epi*-Cubebol	1488	0.1	-	0.2–0.6	0.2–1.1	-
Caryophyllene oxide	1580	0.1–0.6	0.2–2.8	0.5–1.3	0.1–1.1	1.2
1,10-di-epi-cubenol	1612	1.2–7.7	-	-	-	-
T-Muurolol	1642	-	-	0.1–2	1.1	-
β-Eudesmol	1650	0.9	0.4–1.2	-	0.05–0.08	-
α-Cadinol	1651	0.2–4.1	4.2	0.5–5.4	3.5	1.7
**Others**						
3,5-Dimethylene-1,4,4-trimethylcyclopentene	924 ^a^	-	-	2.5–5.8	-	-
1-octen-3-ol	980	0.2	0.02–0.5	0.2	0.06–0.3	-
2,3,4,5-Tetramethyl-2-cyclopenten-1-one	1054 ^b^	-	-	0.2–5.1	-	-
3,4,4-Trimethyl-2-cyclohexanone	1055 ^c^	-	-	0.4–3.1	-	-
2,3,5,5-Tetramethyl-4-methylene-2-cyclopentenone	1152	-	-	0.4–11.4	-	-
1,1,2,3-Tetramethyl-4-hidroxymethyl- 2-ciclopentene	1155 ^b^	-	-	0.1–0.6	-	-
2,3,4,4-Tetramethyl-5-methylcyclopenten-2-enone	1160 ^c^	-	-	2–38.3	-	-
1,1,2,3-Tetramethyl-4-hydroxymethyl-2-cyclopentene	-	-	-	2–3.1	-	-
2-Methoxy-4-vinylphenol	1315	-	-	3	-	-
**Monoterpene hydrocarbons**		0.1–17	0.7–18.9	2.6–6.4	5.2	2.7–19.1
**Oxygenated monoterpenes**		67–87	62–85.4	33–73	75.5	70.8–71
**Sesquiterpene hydrocarbons**		0.5–10	0.6–2.7	1.4–9.3	4.9	0.1–0.3
**Oxygenated sesquiterpenes**		0.3–4.5	0.5–19.8	2.3–12.4	7.4	1.9
**Others**		0.1–0.2	-	2.4–5.1	-	1.0
**Yield (%, *v*/*w*)**		0.5–2	0.8–1	0.7–2.8	0.9–1.3	0.5–1.2

The values are present in % of relative peak area. * RI Lit: Literature Retention Index in a non-polar column: DIMS5P-dimethylsilicone with 5% phenyl groups in [45]. LP [5,6,38,39,42,43,44]; LS [46,47,48,49,50,51,52,53,54,55,56,57,58,59,60,61,62,63,64,65,66,67,68,69]; LSL [5,14,19,20,25,36,63,70,71,72,73,74,75,76]; LSS [40,77,78,79,80,81,82,83]; LV [5,30,84,85]. ^a^ RI consulted in [15]. ^b^ RI consulted in [73]. ^c^ RI consulted in [36].

**Table 2:** Phytochemical composition of polar and non-polar extracts of *Lavandula* sp. section *Stoechas*.

	*L. pedunculata* ^LP^	*L. stoechas* ^LS^	*L. stoechas* subsp. *luisieri* ^LSL^	*L. stoechas* subsp. *stoechas* ^LSS^	*L. viridis* ^LV^
**Hydroxybenzoic acids**					
Gentisic acid		0.001 (M; AP)			
Hydroxybenzoic acid		0.07 (HM;M; AP)	0.02 (W;E; AP)		
4-Hydroxybenzoic acid 4-(6-O-sulfo)glucoside		+ (HM; AP)			
Methyl dihydroxybenzoic		+ (HM; AP)			
Protocatechuic acid		+ (HM;M; AP)	0.01 (W;E; AP)		
Vanillic acid		+ (HM;M; AP)	0.01 (W;E; AP)		
**Hydroxycinnamic acids**					
**Caffeic acid**	0.6–4.4 (W;HE; AP)	0.03–0.9 (M; AP; L; HM;W; AP)	0.04 (W;E; AP)		2.6 (HE; AP)
**Caffeic acid hexoside**	0.03–5 (W;HE; AP)				
Caffeoyl hexoside		+ (HM; AP)			
Caffeoyl feruloyl tartaric acid		+ (HM; AP)			
6-Caffeoylsucrose		+ (HM; AP)			
3-*O*-Caffeoylquinic acid	0.01 (W; F)				0.1–2.5 (HE; AP)
4-*O*-Caffeoylquinic acid	0.1–0.7 (W; HE; F)				1.3–1.8 (HE; AP)
Caftaric acid		+ (HM; AP)			
Chicoric acid		+ (HM; AP)			
Chlorogenic acid	0.01–1.2 (W;HE; F)	+ (HM;M; AP)	0.1 (W;E; AP)	18.5 (HM; AP)	0.6–2.3 (HE; AP)
*p*-Coumaric acid		0.003–0.02 (M; AP; L; HM)			
Coumaric acid hexoside		+ (HM; AP)			
*p*-Coumaroyl hexoside	0.5–4.2 (W;HE; AP)				
Dihydrocaffeic acid		+ (HM; AP)			
3,4-Dihydroxyphenyllactic acid hexoside		+ (HM; AP)			
3,4-Dihydroxyphenyllactic acid		+ (HM; AP)			
3-(3,4-Dihydroxyphenyl)-2-hydroxypropanoic acid		+ (HM; AP)			
Ferulic acid		0.03 (M;HM; AP)	0.2 (W;E; AP)		
Fertaric acid		+ (HM; AP)			
Hydroxyhydrocinnamic acid glucoside		+ (HM; AP)			
Isosalvianolic acid A		+ (HM; AP)			
Lithospermic acid A	2.9–26.2 (W;HE; AP)				
Methyl caffeate		+ (HM; AP)			
Methyl melitrate		+ (HM; AP)			
Methyl rosmarinate		+ (HM; AP)			
Rosmarinic acid	0.01–550 (W;HE; AP;F)	0.08–8.4 (W;M; L; AP;HM; F; AP)	3–73.2 (W;E; AP)	74 (HM;AP)	1.3–38.8 (HE; AP)
Rosmarinic acid hexoside	0.9–3.3 (W;HE; AP)	+ (HM; F; AP)			
Sagerinic acid	1.1–6.6 (W;HE; AP)	+ (HM; AP)			
Salicylic acid		0.001 (M; AP)			
Salvianolic acid A		+ (W;M;HM; F;AP)			
Salvianolic acid B	8.7–582 (W;HE; AP)	+ (W;M; AP;HM; F; AP)			
Salvianolic acid C		+ (HM; AP)			
Yunnaneic acid F		+ (HM; AP)			
**Flavonoids**					
6″-*O*-Acetylgenistin		+ (W;M; F)			
Apigenin	0.8–2.7 (HE; F)	+ (HM; AP)			
Apigenin di-*C*-hexoside		+ (HM; AP)			
Apigenin *C*-hexoside		+ (HM; AP)			
Apigenin O-glucuronide		+ (W;M; F)			
Apigenin 7-*O*-glucoside		+ (M; HM; AP)		4.1 (HM; AP)	
Apigenin 7-*O*-glucuronide		+ (HM; AP)			
Eriodictyol		0.1 (M; L)			
Eriodictyol-*O*-glucuronide	0.1–16.7 (W;HE; AP)				
Genkwanin		+ (M;HM; AP)			
Luteolin	0.01–4.9(W;HE; AP;F)	+ (HM; AP)			0.2–7.1 (HE; AP)
Luteolin 7,4′-di-glucuronide		+ (HM; AP)			
Luteolin 7-*O*-glucuronide	12.2–101.5 (W;HE; AP)	+ (W;HM; F; AP)			
Luteolin 7-*O*-glucoside		+ (W;HM; F;AP)		14.9 (HM; AP)	13.4 (HE; AP)
Luteolin-*O*-hexosyl-*O*-glucuronide	1.4–8.7 (W;HE; AP)				
Methylluteolin-*O*-glucuronide	1.8–19.8 (W;HE; AP)				
Pinocembrin					2.7–12.7 (HE; AP)
Quercetin		1 (M; L)			
Quercetin 3-*O*-glucoside		+ (W;M; F)			
Rutin		0.5 (M; L)			
**Sesquiterpenoids/Triterpenes**					
3-*oxo*-Cadinol			100.3 (E; AP)		
Oleanolic acid			29.5–34.7 (E; AP)		
Stoechanones A e B		+ (HM; AP)			
Tormentic acid			138.5 (E; AP)		
Ursolic acid			124 (E; AP)		
**Others**					
5-Hydroxymethyl-2,3,4,4-tetramethylcyclopent-2-en-1-one			43.3–79.3 (E; AP)		
Esculetin		+ (HM; AP)			
5-Nonadecylresorcinol		+ (W;M; F)			

The values are present in mg/g. LP [6,8]; LS [26,60,69,105,106,107]; LSL [21,25]; LSS [108]; LV [109,110]. M: methanolic extract; HE: hydroethanolic extract; W: water extract; E: ethanolic extract; HM: hydromethanolic extract; AP: aerial parts; F: flowers; L: leaves; + symbol corresponds to the presence of the compound.

**Table 3:** Phenolic and flavonoid content and antioxidant activity of *Lavandula* sp. section *Stoechas* extracts.

	*L. pedunculata* ^LP^			*L. stoechas* ^LS^			*L. stoechas* subsp. *luisieri* ^LSL^			*L. stoechas* subsp. *stoechas* ^LSS^		*L. viridis* ^LV^		
	EO	NPE	PE	EO	NPE	PE	EO	NPE	PE	EO	PE	EO	NPE	PE
Phenolics content (mg GAE/g); ^A^(mg GAE/mL)	50	≈450 to 474	631–1040		227	154 4286 ^A^	≈200 to 1612	67–1778	277–1689		25–82			
Flavonoids content (mg RE/g); ^B^(mg QE/g)	3 to <50	311–314	242–371		40	134	8 to <50 33 ^B^	196–392 7.2–28 ^B^	451–459 13 ^B^		10 35 ^B^			
DPPH scavenging activity IC_50_ (µg/mL)		137	68	221–5100	1.2	1780	8830	123–1323	26–1070	22–23	34–453			
% DPPH inhibition (50 µg/mL); ^C^(60 µg/mL); ^D^(100 µg/mL); ^E^(500 µg/mL); ^F^(1200 µg/mL)	59–60 10 ^D^	67–710 70 ^D^	≈70 to 71 93 ^D^		45 ^C^	50 ^C^ 69 ^E^	67–69	57–64	69–72			56 ^F^		
Lipid peroxidation inhibition IC_50_ (µg/mL)		190	253				1530							
% Lipid peroxidation inhibition 0.06 g/mL); ^G^(0.1 mg/mL); ^H^(0.2 mg/mL); ^I^(0.3 mg/mL)	33 ^G^ 17 ^H^ ≈ 18 ^I^	≈28 ^H^ ≈29 ^I^	20 ^G^ ≈40 ^H^ ≈35 ^I^		97	95	15 ^H^ ≈17 ^I^	≈25 ^H^ ≈25 ^I^	≈49 ^H^ ≈40 ^I^					
TEAC (µmol TE/g)	-	224	866	117									332	671
ORAC (µmol TE/g); ^J^(µmol TE/µL)	157	861	3018	1.7 ^J^								468	1184	1502
TBARS IC50 (µg/mL)		17	14											
FRAP (mg TE/g); ^K^(µmol/g); ^L^(mM FeSO_4_E/g)								52	80	21 ^K^	224 ^K^ 2.6 ^L^			
CUPRAC (mg AAE/g)											370			
Reducing power IC_50_ (µg/mL); ^M^(mg TE/g); ^N^(mg AAE/mL)		67	51	108 5.7 ^N^				52 ^M^	36 ^M^					
Chelating ability IC_50_ (mg/mL); ^O^(mg EDA/mL)				1.8 ^O^						18	3.5			
% Ferrozine complex inhibition (60 µg/mL)					92	84								
% Superoxide generation inhibition (60 µg/mL)					77	78								

LP [6,7,8,128,129]; LS [51,53,55,57,60,65,69,105,127,130,131,132]; LSL [7,20,21,75,128]; LSS [81,108]; LV [30,109]. EO: essential oil; NPE: non-polar extract; PE: polar extract; GAE: gallic acid equivalent; RE: rutin equivalent; QE: quercetin equivalent; DPPH: radical 2,2-diphenyl-1-picryl-hydrazyl; IC_50_: concentration corresponds to 50% of the inhibition of free radicals; TEAC: Trolox equivalent antioxidant capacity assay; TE: Trolox equivalent; FeSO_4_E: iron sulphate equivalent; ORAC: oxygen radical absorbance capacity assay; TBARS: thiobarbituric acid reactive substances assay; FRAP: ferric reducing antioxidant power assay; AAE: ascorbic acid equivalent; ≈ corresponds to approximated values.

**Table 4:** Antimicrobial activity of *Lavandula* sp. section *Stoechas* extracts.

	*L. pedunculata* ^LP^			*L. stoechas* ^LS^			*L. stoechas* subsp. *luisieri* ^LSL^			*L. stoechas* subsp. *stoechas* ^LSS^	*L. viridis* ^LV^
	EO	NPE	PE	EO	NPE	PE	EO	NPE	NPE	EO	EO
**Gram-negative**											
*Acinetobacter baumannii*				0.2	35.9						
*Enterobacter cloacae*		40/75	150/300				+	+			
*Escherichia coli*	5	75/150; 14/14 *	40/150	0.2	+		1000	+		250	
				5 *			22/22				
*Haemophilus influenzae*										1250	
*Klebsiella pneumoniae*	+	250	125	0.2			+	125	250	+	
*Moraxella catarrhalis*										1250	
*Morganella morganii*							500				
*Proteus mirabilis*				10 *			250			+	
*Proteus vulgaris*					35.9						
*Pseudomonas aeruginosa*	20	100/150	150/300	0.2	35.9	250	500	+		250–1250	
*Salmonella enterica*				0.2							
*Salmonella enterica* serovar *Enteritidis*				+	17.9		750				
*Salmonella kentucky*					35.9						
*Salmonella* spp.							22/22				
*Salmonella typhimurium*		100/150	150/300	+	35.9		750	+		250	
*Serratia marcescens*							+	+			
*Yersinia enterocolitica*		14/>56 *			6500	3250					
**Gram-positive**											
*Bacillus cereus*		50/75	75/150	+			+	7670	9830		
*Bacillus subtilis*		4/8 *		0.2	35.9	6500	+	+			
				20 *	6500						
*Enterococcus durans*					35.9						
*Enterococcus faecalis*					35.9		125	62	62		
*Enterococcus faecium*		2/14 *			17.9						
*Listeria innocua*					35.9						
*Listeria monocytogenes*		100/150 0.5/8 *	150/300	2.5 *	35.9						
*Micrococcus flavus*		75/150	150/300								
*Mycobacterium smegmatis*								125	250		
*Staphylococcus aureus*	5	125 20/40 2/14 *	250 40/100	5 *	17.9 3250	125 3250	250 11/11	62	62 9830	31.3 310	
*Staphylococcus epidermidis*					8.9		125	125	62	250	
*Streptococcus fasciens*	5										
*Streptococcus hirae*							+	+			
*Streptococcus pneumoniae*					35.9					620	
*Streptococcus pyogenes*							+	+		620	
**Yeasts**											
*Aureobasidium pullulans*							0.3/2.3 *				
*Candida albicans*	2.5/2.5 *			0.5 2.5/ 2.5 *	35.9		250	62.5		125	1.3/1.3 *
							1.3/2.5*				
*Candida glabrata*										500	
*Candida guillermondii*	62.5	62,5		1.3/1.3 *			62.5				0.6/0.6 *
	1.3/1.3 *						1.3/1.3 *				
*Candida krusei*	1.3/2.5 *			2.5/2.5 *			125			500	1.3/1.3 *
							2.5/2.5 *				
*Candida parapsilopsis*	2.5/2.5 *			2.5/2.5 *			125			500	1.3/1.3 *
							2.5/2.5 *				
*Candida tropicalis*	1.3/2.5 *			2.5/2.5 *			2.5/2.5 *			250	1.3/1.3 *
*Candida utilis*										500	
*Candida zeylanoides*										500	
*Cryptococcus neoformans*	15.5 0.3/ 0.6 *	31	62.5	0.6/0.6*			15.5 0.6/0.6 *	31	62.5	250	0.6/0.6 *
*Rhodotorula rubra*	62.5	62.5							31		
*Saccharomyces cerevisiae*		31	62.5				31		31		
*Saccothecium rubi*							0.3/1.2 *				
*Trichosporon cutaneum*	62.5	31					31		62.5		
**Fungi**											
*Alternaria alternaria*				+							
*Alternaria* spp.				4							
*Aspergillus carbonarius*							0.3/9.3 *				
*Aspergillus flavus*	5/5 *			0.5 5 *			1.3 *			+	5/10 *
*Aspergillus fumigatus*	2.5/5 *	40/75	150/200	1.25 *			0.6/10 *				2.5/5 *
*Aspergillus niger*	5/5 *	40/75	150/200	0.5 2.5 *			15.5	7.5	62.5		2.5/20 *
							0.3/20 *				
*Aspergillus ochraceus*		20/75	150/200								
*Aspergillus versicolor*		30/75	100/200								
*Botryris cinerea*				+							
*Epidermophyton floccosum*	0.3/ 0.3 *			0.3/ 0.6 *			0.2/0.2 *				0.3/0.3 *
*Fusarium oxysporum*				+						+	
*Fusarium oxysporum* f. sp. *lini*				0.2							
*Fusarium* spp.				1.6							
*Geotrichum klebahnii*							125				
*Microsporum canis*	0.3/0.3 *			0.6/0.6 *			0.2/0.2 *				0.3/0.3 *
*Microsporum gypseum*	0.6/0.6 *			0.6/0.6 *			0.3/0.3 *				0.6/0.6 *
*Mucor ramannianus*				0.2							
*Mucor* spp.				4							
*Penicillium brevicompactum*							0.3/2.3 *				
*Penicillium funiculosum*		40/15	200/250								
*Penicillium ochrochloron*		30/40	200/250								
*Penicillium roqueforti*							0.1/0.6 *				
*Penicillium* spp.				4.5							
*Penicillium verrucosum* var. *cyclopium*		50/75	100/200								
*Rhizopus stolonifer*				1.6			0.1/4.7 *				
*Trichoderma* spp.				4							
*Trichoderma viride*		20/20	100/200								
*Trichophyton mentagrophytes*	0.6/0.6 *			0.6/0.6 *			200				0.3/0.6 *
							0.3/0.3 *				
*Trichophyton mentagrophytes* *var. interdigitale*				0.6/1.3 *			0.2/0.3 *				0.3/0.6 *
*Trichophyton rubrum*	0.3/0.3 *			0.6/1.3 *			200				0.3/0.3 *
							0.2/0.3 *				
*Trichophyton verrucosum*				0.6/0.6 *			0.3/0.3 *				0.3/0.3 *

The values are presented in MIC or MIC/MLC (µg/mL or µL/mL *). LP [8,38,43,44,128,138]; LS [46,51,56,57,58,60,62,63,65,86,139,140,141,142]; LSL [7,15,19,20,21,36,63,75,76,128,138]; LSS [40,77,80,82]; LV [85]. EO: essential oil; NPE: non-polar extract; PE: polar extract. The + symbol represents the action reported by the inhibition halo.
